# Time course change of COX2-PGI_2_/TXA_2_ following global cerebral ischemia reperfusion injury in rat hippocampus

**DOI:** 10.1186/1744-9081-10-42

**Published:** 2014-11-11

**Authors:** Lijuan Yu, Bin Yang, Jia Wang, Lei Zhao, Weinan Luo, Qingsong Jiang, Junqing Yang

**Affiliations:** Department of Pharmacology, Chongqing Key Laboratory of Biochemistry and Molecular Pharmacology, Chongqing Medical University, Medical College Rd. No 1, Chongqing, 400016 China

**Keywords:** Global cerebral ischemia reperfusion, PGI_2_, TXA_2_, COX2, PGI_2_/TXA_2_, Neuroinflammation

## Abstract

**Background:**

Neuroinflammation plays pivotal roles in the progression of cerebral ischemia injury. Prostaglandins (PGs) as the major inflammatory mediators in the brain participate in the pathophysiological processes of cerebral ischemia injury. Cyclooxygenase-2 (COX2) is the rate-limiting enzyme of PGs, and thus it is necessary to characterize of the expression patterns of COX2 and its downstream products at the same time in a cerebral ischemia/reperfusion (I/R) model.

**Methods:**

The levels of prostacyclin (PGI_2_) and thromboxane (TXA_2_) and the expression of COX2 were detected in the rat hippocampus at different time points after reperfusion (30 min, 2 h, 6 h, 24 h, 48 h, 7 d, and 15 d).

**Results:**

The COX2 mRNA and protein expressions in hippocampus both remarkably increased at 30 min, and peaked at 7 d after global cerebral I/R compared with the sham-operated group. The level of PGI_2_ significantly increased at 2 h after reperfusion, with a peak at 48 h, but was still significantly higher than the sham-operated animals at 15 d. TXA_2_ level decreased at 30 min and 2 h after reperfusion, but significantly increased at 6 h and peaked at 48 h. PGI_2_/TXA_2_ ratio increased at 30 min after reperfusion, and peaked at 48 h compared with the sham-operated animals.

**Conclusions:**

I/R injury significantly increased the COX2 expression, PGI_2_ and TXA_2_ levels, and the PGI_2_/TXA_2_ ratio in rat hippocampus in a time-dependent manner. As a consequence, the increased PGI_2_ level and PGI_2_/TXA_2_ ratio may represent a physiological mechanism to protect the brain against the neuronal damage produced by I/R injury.

## Introduction

Cerebral ischemic injury with high morbidity, disability and mortality worldwide has brought heavy psychological burden and economic pressures to patients and the society [1]. It is therefore necessary to sufficiently understand the pathophysiologic mechanisms and effective neuroprotective strategies involved in cerebral ischemia injury.

Cerebral ischemia is defined as a temporal or permanent reduction in cerebral blood flow (CBF) that is insufficient to meet the functional or metabolic demand by the central nervous system (CNS). Reperfusion can restore CBF, but can also exacerbate brain injury. Increasing evidence has recently accumulated to support the notion that oxidative stress and neuroinflammation play pivotal roles in the progression of cerebral ischemia injury [2, 3]. Inflammation is at least partially mediated by prostaglandins (PGs), which are mediated by the rate-limiting enzyme cyclooxygenase (COX). A huge amount of free arachidonic acid (AA) is released from membrane phospholipids after the ischemic event, and then COX catalyzes the conversion of AA into the intermediate PGH_2_, which is then metabolized by cell-specific synthases to produce five PGs: prostacyclin (PGI_2_), thromboxane A_2_ (TXA_2_), PGE_2_, PGF_2α_, and PGD_2_. The five PGs bind to classes of G protein-coupled receptors designated as IP, TP, EP (EP1, 2, 3, and 4), FP and DP (DP1 and 2) to elicit their specific bio-effects on cyclic adenosine monophosphate (cAMP), phosphatidylinositol turnover, and intracellular calcium mobilization, respectively [4]. Some PGs are pro-inflammatory mediators, but others are anti-inflammatory [5]. There are two forms of COX: COX-1 and COX2. COX-1 is expressed constitutively throughout the gastrointestinal system, kidneys, vascular smooth muscle, and platelets, whereas COX2 is an inducible form that contributes to fundamental brain functions, such as synaptic activity, memory consolidation and functional hyperemia under normal conditions in the CNS [6], but its expression can be induced by a variety of stimuli, including bacterial lipopolysaccharide (LPS), pro-inflammatory cytokines, growth factors, and tumor promoters [7]. COX2 participates in the neurodegenerative disease in a PGs-dependent manner [8]. Inhibition of COX2 either pharmacologically or genetically decreases neuronal injury after cerebral ischemia [9, 10]. However, long-term clinical trials show that chronic blockade of COX in patients taking COX2 inhibitors leads to an increased risk of cerebrovascular and cardiovascular complications [11]. Therefore, it is necessary to better understand the COX-modulated downstream pathway. Efforts have been made to elucidate the neurotoxicity of COX2 with the hope of developing new therapeutic agents that avoid the detrimental side effects but still retain the beneficial effects. A better strategy for treating cerebral ischemia injury may focus on modulating the PGs, downstream of the COX pathway [12, 13].

PGI_2_ and TXA_2_, which derive from PGH_2_ via the action of prostacyclin and thromboxane A_2_ synthetase, respectively, are important members of the PG family. The balance between PGI_2_ and TXA_2_ plays a major role in regulation of CBF in response to ischemia [14]. PGI_2_ analogs and TXA_2_ antagonists are potentially useful for the treatment in different ischemia animal models by improving CBF [15, 16]. More attention has been paid to the roles of PGI_2_ and TXA_2_ in regulation of blood coagulation and vascular tone [17, 18], but rarely to their effects on neurons. In physiological conditions, PGI_2_ and TXA_2_ are highly expressed in the rat cortex and hippocampus [19]. Focal cerebral ischemia reperfusion (I/R) will lead to the expression of prostacyclin synthase in the pyriform cortex, peri-infarct cortical area, subcortical, and CA1 pyramidal neurons [20]. Takechi et al. [21] found a novel subtype of IP receptor which is expressed in neurons but not glial cells in the CNS and is clearly distinct from the peripheral subtype in terms of ligand specificity. TXA_2_ also participates in the progress of fibrillar Aβ-induced extrapyramidal motor dysfunction in rat striatum [22]. Their expression manner and signaling pathways in the CNS are still unclear. The roles of PGI_2_ and TXA_2_ in ischemic brain injury are not yet fully elucidated.

Since PGI_2_ and TXA_2_ participate in the pathophysiological processes of cerebral ischemia injury and COX2 is their rate-limiting enzyme, it is necessary to characterize of their expression patterns at the same time in a cerebral I/R model. The COX2 expression or the levels of PGs have been extensively investigated in different cerebral ischemia animal models, such as the temporal and topographic profiles of COX2 expression during 24 h of focal brain ischemia in rats [23], and the levels of TXA_2_ and PGI_2_ in rat CA1 hippocampus within 4 h after global cerebral I/R with microdialysis probes [24]. However, their observation periods are relatively short, and since the effect of cerebral ischemia on rat is long lasting, observation of COX2 or its downstream alone cannot reflect their interrelation. To the best of our knowledge, the temporal expression profiles of COX2 and its downstream have not been systematically reported at the same time in any ischemia model.

In this study, we will establish a global cerebral I/R rat model to investigate the time course of COX2 expression, PGI_2_ and TXA_2_ levels, and the ratio of PGI_2_/TXA_2_ in the rat hippocampus at different time points after global cerebral I/R. The results may provide important insights into the mechanisms of ischemic brain injury and provide therapeutic benefits in the future.

## Experimental procedures

### Animal groups

All experiments were performed following the *Guide for the Care and Use of Laboratory Animals* and were approved by the Animal Care and Use Committee at Chongqing Medical University. A total of 120 pathogen-free male wistar rats with body weight of 200–250 g were used. The animals were housed five per cage at room temperature of 20 ± 2°C, 55 ± 10% humility, and light on 12 h/d (08:00–20:00) with ad libitum access to food and water. All animals were fasted for 12 h before surgery and returned to the preoperative conditions until sacrifice. The rats were randomly divided into 8 groups (*n* = 15 each): a sham-operated group, and 7 treated groups tested at 30 min, 2 h, 6 h, 24 h, 48 h, 7 d, and 15 d after I/R respectively. From each group, 4 rats were used for determination of cerebral edema, 3 rats for histopathological examination, 4 rats for measurement of SOD activity, MDA content, and PGI_2_ and TXA_2_ levels, and 4 rats for detection of COX2 mRNA and protein expressions in the hippocampus.

### Establishment of global cerebral ischemia model

The bilateral common carotid arteries were occluded for 20 min combined with systemic hypotension and then made in rats to establish a global cerebral I/R model [25]. Briefly, the rats were anaesthetized with 4% chloral hydrate (10 mL/kg, ip.) and in the dorsal position, the neck area was shaved; both common carotid arteries and the right common carotid vein were exposed carefully by blunt dissection; after the right common carotid vein was ligated at the distal end, a V-shaped incision was made at the distal end. Then 2 mL of heparinized saline infusion (1 ml/100 g, 100 ml of saline containing 250 U heparin) was injected into the vein, 30% of total blood volume of a rat was extracted from the right common carotid vein, and the bilateral common carotid arteries were clamped with microaneurysm clips for 20 min. Then the clips were removed, the extracted blood was reinfused, and the incisions were sutured carefully. In sham-operated animals, both common carotid arteries were exposed but not occluded and blood was not withdrawn from the carotid vein. The rectal temperature was controlled carefully at 37 ± 0.5°C during the experiment using a heating lamp and a heating pad.

### Morris water maze test

Morris water maze was used to investigate the rats’ spatial learning and memory functions in the laboratory. The maze was a round tank (150 cm in radius and 60 cm in height) filled with water at about 24°C. The tank was divided into 4 equal quadrants (A - D). An invisible platform (25 cm in diameter) was submerged 2 cm below the water surface and placed at the midpoint of one quadrant. The test was carried out according to a previous protocol [26]. The rats were subjected to a training phase (day 8 – 11 after I/R) and a testing phase (day 12 after I/R). The animals were trained for 4 consecutive days at about the same time (8:00 – 11:00 pm) with 4 trials each day. In each trial, the rats were left in a new quadrant facing the wall and allowed to swim freely to find the escape platform. If a rat failed within a time limit of 180 s, it was guided to the platform by the experimenter. If the rat succeeded, it was allowed to stay on the platform for 10 s before the next animal was tested. The test session was performed once on the testing phase, the platform was removed from the tank, and the rat was permitted to swim in the Morris water maze until it located the platform. A video camera above the water maze pool was linked to a computer, and recorded the time of escape latency and the length of swimming path.

### Histopathologic examination

After deep anesthesia with 4% chloral hydrate (400 mg/kg, ip), the rats were transcardially perfused with 200 ml of cold saline, followed by 250 ml of phosphate buffer solution (PBS, 0.1 M; pH 7.4) containing 4% paraformaldehyde. Then the brains were removed, and the middle part of the brain was immersed in 4% paraformaldehyde and kept at 4°C overnight, and the coronal brain was cut to 5 μm thick sections. For each animal, 4 sections containing the dorsal hippocampus were mounted on paraffin-coated slides and stained with hematoxylin-eosin to examine the integrity of the CA1 pyramidal cell layers [27]. Hippocampus neuronal damage in the dorsal hippocampal CA1 subfield was detected at 400×. Pyramidal cells with a distinct nucleus and nucleolus were regarded as intact, while neurons showing typical signs of dark staining, shrinkage and dysmorphic shape were considered as damaged. The number of necrotic neuronal injured cells was counted and expressed as percentage for each hippocampal region.

### Measurement of SOD activity and MDA level

SOD activities and MDA content were assessed with relevant detection kits (Nanjing Jiancheng Bioengineering Institute, China), using xanthine oxidase method and thiobarbituric acid method respectively [28]. Briefly, 10% hippocampus homogenate was prepared in ice-cold physiological saline solution (0.9%), and after centrifugation at 4000 × g for 10 min, the supernatant was collected and tested according to the manufacture’s directions.

### Measurement of 6-keto-PGF_1α_ and TXB_2_ levels

Because both PGI_2_ and TXA_2_ have a very short half-life and are ready to convert to 6-keto-PGF_1α_ [29, 30] and TXB_2_ [31] respectively, we then measured 6-keto-PGF_1α_ and TXB_2_ levels. Briefly, hippocampus tissues were homogenized in a buffer containing 1 mM EDTA, 10 uM indomethacin, and 0.1 M PBS. Homogenates were centrifuged at 12000 × g and 4°C for 15 min. Supernatants were removed and assayed in triplicate using a 6-keto-PGF_1a_ and TXB_2_ enzyme linked immunosorbent assay (ELISA) kit according to the manufacturer’s guidelines (Cayman Chemical).

### Reverse transcriptase-polymerase chain reaction (RT-PCR)

The primers for COX2 and β-Actin were chosen from sequence in Genbank and synthesized by Sangon Biotech Co., Ltd. (Shanghai, China). The COX2 primers were: forwards, 5′-CACGGACTTGCTCACTTTGT-3′; reverse, 5′-GAACGCTTTGCGGTACTCAT-3′, which result in a 162-bp PCR product. The β-Actin as an internal reference primer was: forwards, 5′-AGAGGGAAATCGTGCGTGAC-3′; reverse, 5′-GTGCTAGGAGCCAGGGCAGTA-3′, which result in a 358.6 bp PCR product. PCR was performed as follows: initial denaturation at 94 for 4 min; 35 cycles of 94°C for 15 s, 57.6 for 15 s, and 72 for 40 s; then 72 for 5 min for the final extension. PCR products were separated by electrophoresis using 2% agarose-0.5 × TAE gels containing 0.05 ug/ml ethidium bromide. The band intensity was analyzed densitometrically using Quantity One (Bio-Rad Laboratories, USA). Quantities of PCR product were normalized by dividing the average gray level of the β-actin amplication. In this experiment, 4 samples were all tested 3 times, and the average gray level was taken.

### Western blot analysis

Equal amounts of samples (20 μg of protein) were separated on 10% sodium dodecyl sulfate polyacrylamide gel electropheresis (SDS-PAGE) and transferred to polyvinylidene difluoride membranes. The membranes were incubated with 5% w/v dried fat-free milk in 0.05% Tween-20 in Tris-buffered saline and Tween 20 (TBST) at room temperature for 2 h. Then the membranes were incubated with a primary rabbit anti-rat COX2 antibody (1:100) at 4°C overnight. The second day the blots were washed with TBST (10 min × 3) and exposed to the secondary antibody (1:2000) at room temperature for 1 h. The membranes were washed again with TBST (10 min × 3) and revealed with an enhanced chemiluminescence system (ECL kit; Pierce Biotechnology). The expression in each sample was analyzed with Quantity One (BioRad) and quantified with the ratio to β-Actin. In each experiment, 4 samples were assayed in triplicate, respectively, and the average value was taken.

### Immunohistochemistry

Immunohistochemistry was performed to investigate the expression of COX2 in the rat hippocampus. Briefly, hippocampus sections of 3 rats from each group were dewaxed and rehydrated in decreasing concentrations ethanol. Then the slides were blocked for endogenous peroxidase in 3% H_2_O_2_ in methanol for 20 min at room temperature. Slides were washed with PBS for several times and pre-incubated in 1% BSA for 30 min at room temperature. Thereafter, the slides were incubated with anti-COX2 polyclonal rabbit (dilution 1:100) at 4°C overnight. Then, the sections were incubated with biotinylated secondary antibody (dilution 1:500) for 30 min at 37°C. Finally, the color was developed with 3, 30-diaminobenzidine (DAB) reagent for 2 min. Images were observed using an Olympus microscope system (Olympus, Tokyo, Japan).

### Statistical analysis

The results of each group are expressed as mean ± standard deviation (S.D.). Statistical analysis was analyzed with SPSS16.0 (IBM, USA). Differences between groups were tested with one-way analysis of variance (ANOVA). Further comparisons among groups were made according to post-hoc test. P <0.05 was considered as significant.

## Results

### Effects of global cerebral ischemia on CBF in rats

**Figure 1:**
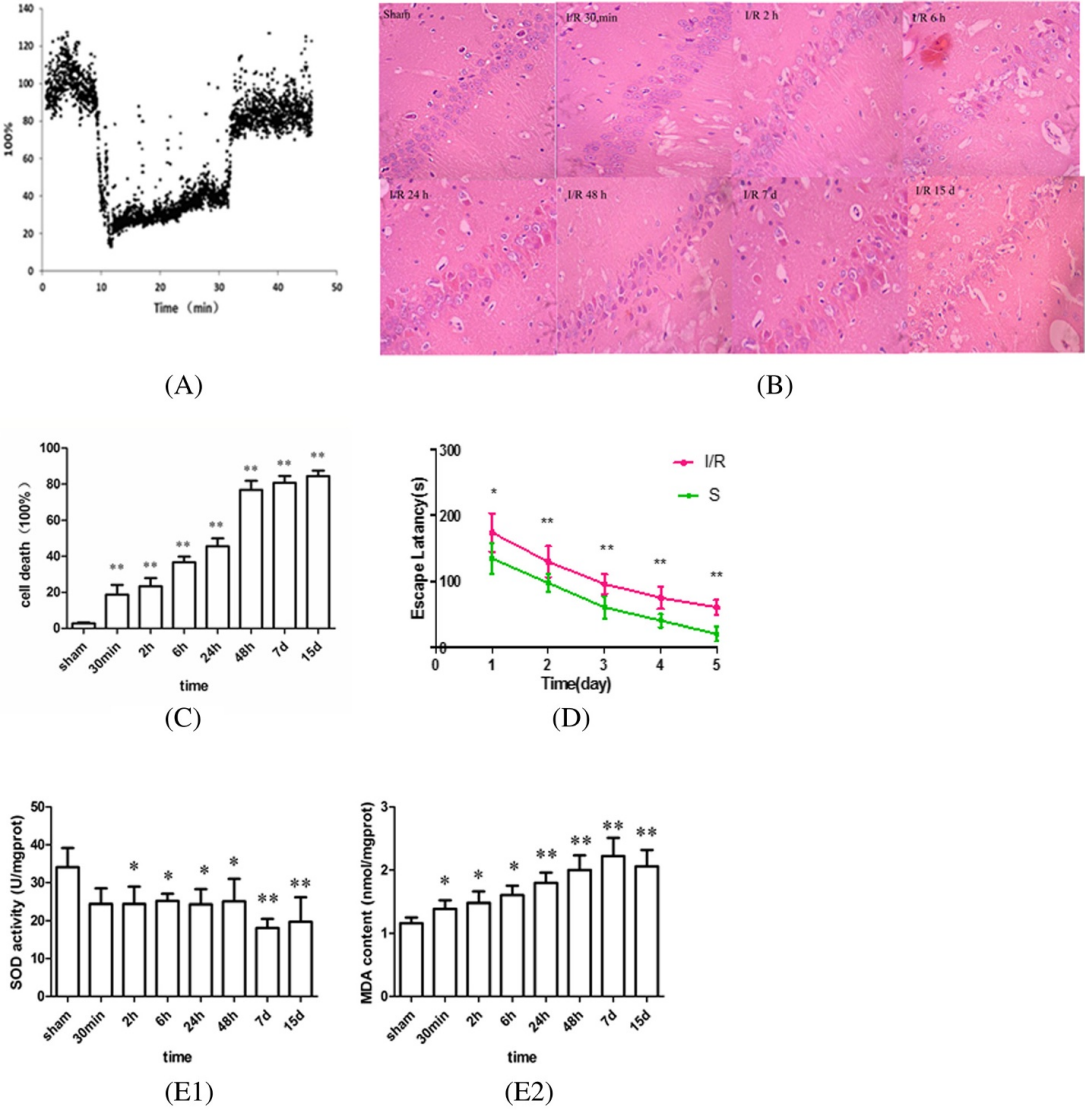
**Change of cerebral blood flow, morphology, cell death rate, escape latency, SOD activities and MDA contents in I/R rat. (A)** Average cerebral blood flow measured before, during, and after global cerebral ischemia in rats. The level of CBF before ischemia was set to 100%. CBF decreased rapidly to 30% of the baseline during the ischemia phase and increased to 75% of the pre-ischemic level during the reperfusion phase. **(B)** Effects of I/R on morphological change of rat hippocampal neurons. Representative microscopic photographs of hematoxylin-eosin–stained rat hippocampal CA1 neurons at different reperfusion time points following 20 min of global ischemia. Sections are show at 400× power. **(C)** Group data showing the cell death rate. Normal neurons in sham operated group is well structured, closely arranged, neurons in ischemia group showed obviously karyopyknosis and swelling, the injury aggravated along with the reperfusion and peaked at 15 d. **(D)** The escape latency of rats in the sham and operated groups. I/R rats had a significantly longer mean latency to find the platform compared with the sham group. **(E)** SOD activities **(E1)** and MDA contents **(E2)** in the hippocampus at the different reperfusion time after 20 min of global ischemia in rats. SOD activity significantly decreased after I/R injury, and reached maximal reduction at 7 d, MDA content progressively increased from 30 min to 15 d after and reached a peak level at 7 d. (Compared with the sham group, ^*^P < 0.05, ^**^P < 0.01).

CBF was measured with a moorVMS-LDF laser Doppler blood flow monitor during the operation phase. When the common carotid arteries were clamped, CBF decreased rapidly to 30% of the baseline within seconds. Upon the release of carotid ligatures, CBF started to increase to 75% of the pre-ischemic level within seconds (Figure 1A).

### Morphological changes of rat hippocampus at different time points after reperfusion

The sham-operated rats exhibited normal neurons with well-structured, closely-arranged, clear and intact cellular form. Microscopic results showed obvious neuronal injury following cerebral ischemia. Karyopyknosis and swelling were observed in neurons from the ischemic rats (Figure 1B). And the number of dead cells increased in the hippocampal CA1 subfield in I/R rats (Figure 1C).

### Effects of I/R on spatial learning and memory functions in rats

At the training phase, the escape latency in all of the groups tended to decrease over time. The escape latency increased significantly in the I/R group compared to the sham-operated group in the Morris water maze at both the training phase and the test phase (Figure 1D).

### Effects of global cerebral ischemia on superoxide dismutase (SOD) activity and malondialdehyde (MDA) content in hippocampus

The SOD activities and MDA content changed dynamically during the 15-d period. In the hippocampus, SOD activities significantly decreased (P < 0.05) in the early reperfusion periods (2, 6, 24, and 48 h) following global cerebral ischemia, and minimized at 7 d (P < 0.01) compared with the sham-operated group (Figure 1E1). By contrast, the MDA content progressively increased from 30 min to 15 d after global cerebral ischemia and peaked at 7 d (P < 0.01) (Figure 1E2).

### Levels of 6-keto-PGF_1α_ and TXB_2_ and the 6-keto-PGF_1α_/ TXB_2_ ratio in rat hippocampus at different time points

**Figure 2:**
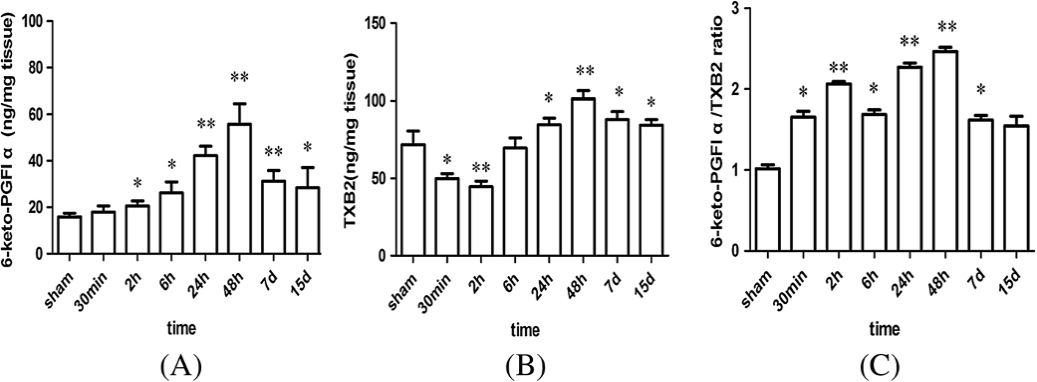
**Levels of 6-keto-PGF**_**1α**_ **(A), TXB**_**2**_ **(B) and 6-keto-PGF**_**1α**_ **/TXB**_**2**_ **ratio (C) in rat hippocampus at the different reperfusion time.** (Compared with the sham group, ^*^P < 0.05, ^**^P < 0.01).

After global ischemia for 20 min, the 6-keto-PGF_1α_ level in rat hippocampus increased significantly at 2 h, with a peak at 48 h, thereafter gradually decreased at 7 d and 15 d, but was still significantly higher than the sham-operated animals (Figure 2A). The level of TXB_2_ significantly decreased at 30 min and 2 h after global ischemia, thereafter began to increase at 6 h (P >0.05) and peaked at 48 h compared to the sham-operated group (Figure 2B). The 6-keto-PGF_1α_/TXB_2_ ratio in rat hippocampus increased as early as 30 min after reperfusion, remained increasing until 2 h, decreased at 6 h, but significantly increased at 24 h and 48 h, and then decreased again at 7 d compared with the sham-operated animals (Figure 2C).

### The effects of I/R on COX2 mRNA and protein (by WB) expressions in rat hippocampus

**Figure 3:**
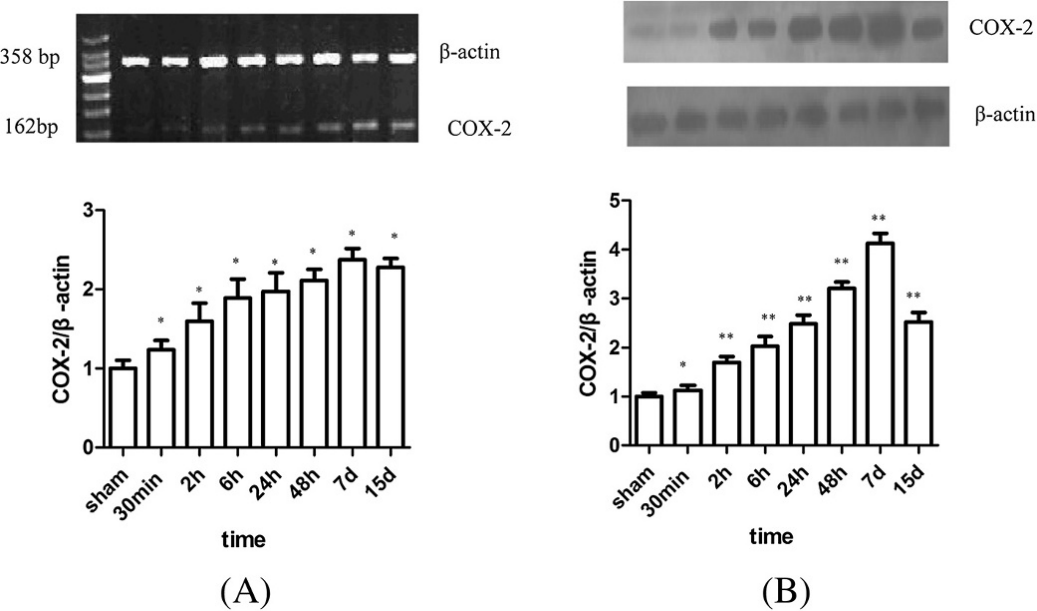
**Time course change of COX2 mRNA (A) and protein (B) expression in I/R rat hippocampus.** COX2 mRNA and protein remarkably increased at 30 min, and reached the peak at 7 d after I/R injury when compared with that of sham operation group. (Compared with the sham group, ^*^P < 0.05, ^**^P < 0.01).

Low levels of COX2 PCR products were observed in the hippocampus of sham-operated rats. The COX2 mRNA level in the hippocampus significantly increased at 30 min and peaked at 7 d, and thereafter decreased at 15 d, but was still significantly higher compared with the sham-operated group (Figure 3A). Then Western Blot was applied to determine whether upregulation of COX2 mRNA resulted in increased synthesis of COX2 protein. As a result, the COX2 protein expression in the rat hippocampus was similar to the COX2 mRNA expression. The COX2 protein expression peaked at 7 d after I/R and decreased at 15 d (Figure 3B).

### Expression of COX2 protein (by immunohistochemistry) in rat hippocampus at different time points after reperfusion

**Figure 4:**
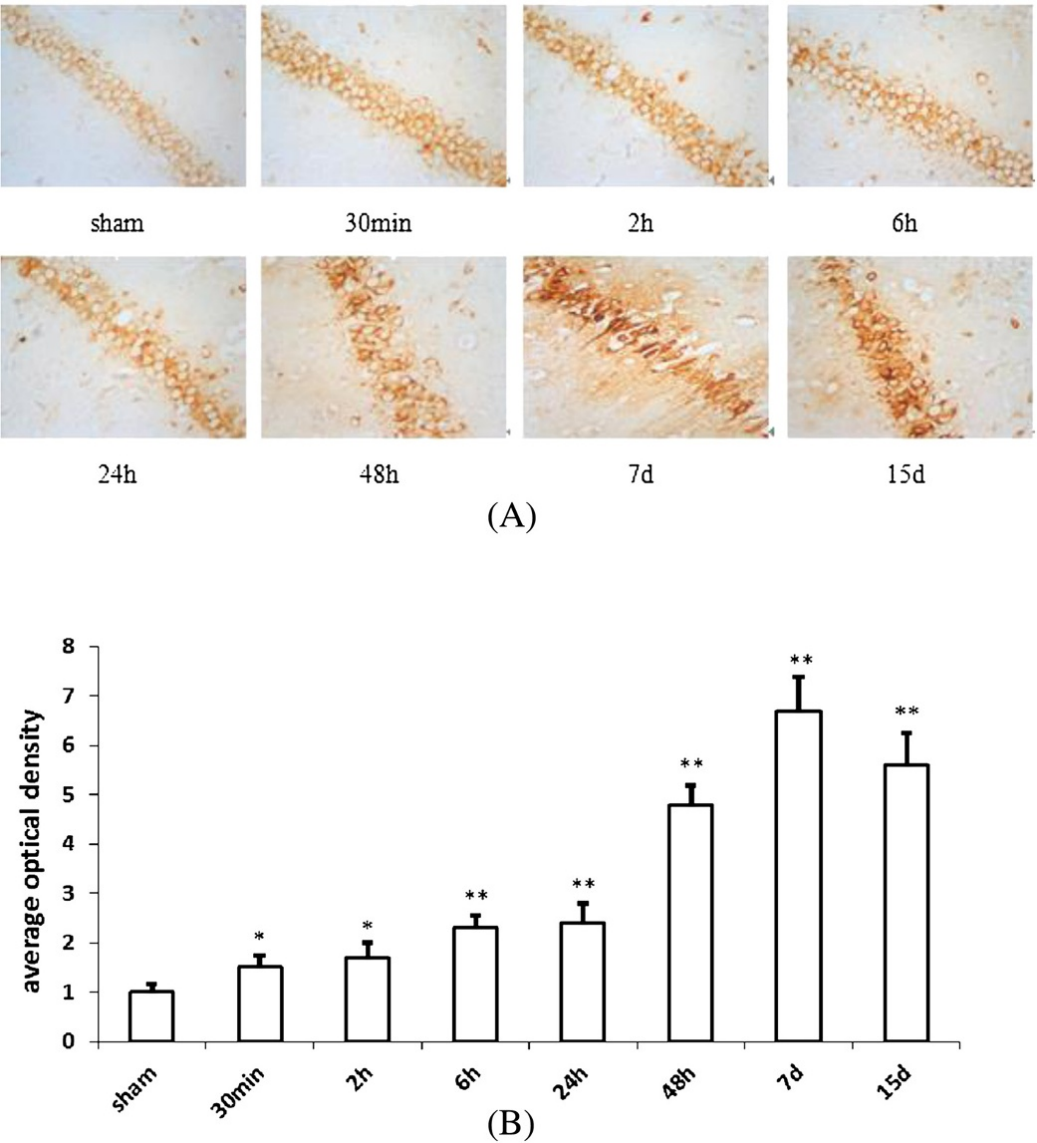
**Time course change of COX2 protein expression in hippocampus of I/R rat. (A)** Time course change of COX2 protein expression in I/R rat hippocampus. **(B)** Group data showing the time course change of COX2 protein expression. Compared with that of sham operation group, COX2 protein remarkably increased at 30 min after I/R, and reached the peak at 7 d after I/R. (Compared with the sham group, ^*^P < 0.05, ^**^P < 0.01).

The expression of COX2 was in the cytoplasm. In the sham group, there was only a faint expression of COX2 in CA1, but COX2 expression increased rapidly in hippocampus CA1 following global ischemia. There was no significant increase of number of cells that express COX2, but an increase of expression in the same population of cells (Figure 4A). The COX2 in the CA1 significantly increased at 30 min (P < 0.05) and peaked at 7 d (P < 0.01), and thereafter decreased at 15 d, but was still significantly higher compared with the sham-operated group (P < 0.01) (Figure 4B).

## Discussion

Oxidative stress has emerged as a key deleterious factor in brain I/R [32]. Ischemic brain injury leads to the overproduction of free radicals [33], which damage cell membranes as well as DNA and proteins by initiating lipid peroxidation [34]. Brain in particular is highly susceptible to free radical-mediated insult, owing to its high content of unsaturated fatty acids and low levels of protective antioxidants. SOD which is a specific scavenger of superoxide anion is involved in the regulation of antioxidant defenses by catalyzing the dismutation of superoxide anion into H_2_O_2_ and O_2_ [35]. MDA is one end product and the biomarker of lipid peroxidation [36]. The SOD activities and MDA contents reflect the balance between oxidative and antioxidative levels in the brain. Our experiments showed a sustained elevation of MDA content and a steady decrease of SOD activity following I/R injury. Our findings are consistent with previous findings, such as that the increased level of lipid peroxidation persists for several days following a brief forebrain ischemia in the gerbil hippocampus [37]. These results together show that ischemic brain injury will lead to the overproduction of free radicals if not cleared timely, and will disturb the balance between oxidative and antioxidative levels in the brain.

Accumulated evidence suggests that the enhanced COX2 participates in the development of neuronal death and in brain ischemia and neurodegenerative diseases [38, 39]. Zhao et al. [40] found that the number of COX2 immunostained cells increased at 12 h, peaked at 48 h, and still elevated until 7 d after focal cerebral ischemia in rats, and COX2 was localized mainly in neurons. COX2 promoted the generation of free radicals when catalyzing AA to PGs, which is associated with the neurontoxicity of COX2 in cerebral ischemia [41]. Oxidative stress can directly induce the transcription of neuronal COX2 [42], which could lead to a vicious cycle associated with neurotoxicity. Our results demonstrated that the time-dependent upregulation of COX2 mRNA and the production of COX2 protein in rat hippocampus persisted for 7 d after ischemia. It suggested that increase of COX2 expression may involve in the brain injury.

The reasons lied in the increased risk of cerebrovascular and cardiovascular complications in patients after long-term take of COX2 inhibitors are that COX2 inhibitors selectively inhibit PGI_2_ in the blood vessel wall without the concomitant inhibition of TXA_2_, and could promote hypertension and thrombosis, and increase cardiovascular risks [43]. Our results demonstrated an upregulation of COX2 expression and an upregulation of PGI_2_ and TXA_2_ levels at 20 min after global ischemia in rat hippocampus. It is reported that increased COX2 expression is associated with increased PGI2 and TXA_2_ levels [44]. PGI_2_ level significantly increased in rat hippocampus at 2 h with a peak at 48 h. Levels of TXA_2_ significantly decreased at 30 min and 2 h, thereafter began to increase at 6 h and peaked at 48 h. Similarly, Huttemeier et al. [24] found that the levels of PGI_2_ and TXA_2_ in the extracellular fluid increased significantly from 40 to 80 min and from 20 to 80 min after global cerebral I/R, respectively. It is unclear why the level of TXA_2_ decreased in the early period following I/R insult. Interestingly, the peak time is 7 d for COX2 expression and is 48 h for its downstream PGI_2_ and TXA_2_. The reason is unclear and should be explored in the future.

Regarding their effects on cerebral I/R injury, the PGI_2_ and TXA_2_ bind to G protein-coupled receptors to exert different bio-effects. The infarct volumes and neurological deficit scores were significantly greater in IP^−/−^ mice than in wildtype mice following focal cerebral ischemia [45]. Activation of IP receptor can attenuate anatomical and functional damages following ischemic stroke [45]. TXA_2_ receptor antagonist can ameliorate brain edema and cerebral infarct areas following focal cerebral ischemia [46], indicating that IP receptor is neuroprotective, while the TP receptor mediates harmful effects on cerebral ischemia injury.

The increased ratio of PGI_2_/TXA_2_ is valuable for neuron-protection. It is reported that a favorable PGI2/TXA2 ratio could protect the brain from injury [20, 47]. PGI_2_/TXA_2_ ratio in rat hippocampus significantly increased from 30 min to 7 d after I/R injury compared with the sham-operated animals. The increased ratio may represent a physiological mechanism to protect the brain against the neuronal damage produced by I/R injury. Similarly, the levels of PGI_2_ and TXA_2_ and the ratio of PGI_2_/TXA_2_ were significantly higher in rat hippocampal slices of 18- and 24-month-old animals compared to 3-month-old animals, probably contributing to protecting the brain against aging-induced neuronal damage [48].

TP receptor antagonist and PGI_2_ alleviate brain injury by regulating collateral blood flow through vascular endothelium TP and IP receptors, respectively [46, 49]. Xiao et al. [50] reported that the size of myocardial infarct in IP^−/−^ mice was significantly larger than that in wild-type mice after cardiac I/R injury, but there was no such difference between TP^−/−^ and wild-type mice, indicating that the endogenous PGI_2_ attenuates I/R injury by acting directly on the cardiac tissue, regardless of its inhibitory effects on blood constituents. In the CNS, prostacyclin synthase was induced in the pyriform cortex, peri-infarct cortical area, subcortical, and CA1 pyramidal neurons following focal cerebral ischemia, which led to increased production of neuron PGI_2_ [20]. The 15R-TIC, a specific ligand for CNS-type PGI_2_ receptor, protects hippocampal CA1 pyramidal neurons against the ischemic delayed neuronal damage in gerbils [51]. The 15R-TIC also has a potent neuroprotective effect against focal cerebral ischemia in a monkey middle cerebral artery occlusion (MCAO) model [52]. PGI_2_ has also been proposed as an agonist of the endogenous PPARδ (also known as PPARβ), and it can act through endogenous PPARδ as a second signaling pathway that controls cell fate [53]. However, Fauti et al. [54] argued that PGI_2_ is insufficient for PPARδ activation and is not a PPARδ agonist in vivo. In the CNS, TXA_2_ receptor is expressed at mRNA level in neurons, glia, and brain stem in normal rats [55]. Thromboxane A_2_ receptor mediates glial morphological change due to activation of RhoA mainly by G_12/13_ [56]. TXA_2_ can elicit stronger vagal or parasympathetic reflexes in rabbits when released during tissue trauma depending on the location [57]. TP receptors play a role in stimulation of afferent neurons and activation of important cardiovascular and pulmonary vagal reflexes [58]. Unilateral injection of Aβ-enhanced apomorphine-induced circling ipsilaterally in rats and significantly elevated the level of TXA_2_, but TXA_2_ receptor antagonists attenuated Aβ-enhanced circling behavior, and TXA_2_ mediated the fibrillar Aβ-induced impairment of striatal motor function [22]. TXA_2_ also mediates iron-overload cardiomyopathy by the TNF-α-associated calcineurin-nuclear factor of activated T cells (NFAT) signaling pathway [59]. The effects of TXA_2_ in the CNS are most independent of its vascular tone, which has received much more attention. PGI_2_ and TXA_2_ are involved in adjustment of I/R injury by acting on the corresponding receptors, but all those remain to be confirmed in the future.

Our study shows that although the level of PGI_2_ and ratio of PGI_2_/TXA_2_ in the hippocampus were significantly induced, the rats displayed obvious spatial learning and memory dysfunctions and obvious hippocampus neurons damage, probably because the increased PGI_2_ level and PGI_2_/TXA_2_ ratio were insufficient to antagonize the I/R-caused damage. Therefore, exogenous PGI_2_ agonists or TXA_2_ antagonists should be supplied. The PGI_2_/TXA_2_ balance may play an important role in protecting the brain from injury. All these need to be confirmed in the future.

## References

[1] Paul SL, Srikanth VK, Thrift AG (2007). The large and growing burden of stroke. Curr Drug Targets.

[2] Ma M, Uekawa K, Hasegawa Y, Nakagawa T, Katayama T, Sueta D, Toyama K, Kataoka K, Koibuchi N, Kuratsu J, Kim-Mitsuyama S (2013). Pretreatment with rosuvastatin protects against focal cerebral ischemia/reperfusion injury in rats through attenuation of oxidative stress and inflammation. Brain Res.

[3] Mehta SL, Manhas N, Raghubir R (2007). Molecular targets in cerebral ischemia for developing novel therapeutics. Brain Res Rev.

[4] Woodward DF, Jones RL, Narumiya S (2011). International Union of Basic and Clinical Pharmacology. LXXXIII: classification of prostanoid receptors, updating 15 years of progress. Pharmacol Rev.

[5] Hata AN, Breyer RM (2004). Pharmacology and signaling of prostaglandin receptors: multiple roles in inflammation and immune modulation. Pharmacol Ther.

[6] Yang H, Chen C (2008). Cyclooxygenase-2 in synaptic signaling. Curr Pharm Des.

[7] Minghetti L (2004). Cyclooxygenase-2 (COX2) in inflammatory and degenerative brain diseases. J Neuropathol Exp Neurol.

[8] Manev H, Chen H, Dzitoyeva S, Manev R (2011). Cyclooxygenases and 5-lipoxygenase in Alzheimer's disease. Prog Neuropsychopharmacol Biol Psychiatry.

[9] Gaur V, Kumar A (2012). Effect of nonselective and selective COX2 inhibitors on memory dysfunction, glutathione system, and tumor necrosis factor alpha level against cerebral ischemia reperfusion injury. Drug Chem Toxicol.

[10] Sasaki T, Kitagawa K, Yamagata K, Takemiya T, Tanaka S, Omura-Matsuoka E, Sugiura S, Matsumoto M, Hori M (2004). Amelioration of hippocampal neuronal damage after transient forebrain ischemia in cyclooxygenase-2-deficient mice. J Cereb Blood Flow Metab.

[11] Khan M, Fraser A (2012). COX2 inhibitors and the risk of cardiovascular thrombotic events. Ir Med J.

[12] Wei G, Kibler KK, Koehler RC, Maruyama T, Narumiya S, Doré S (2008). Prostacyclin receptor deletion aggravates hippocampal neuronal loss after bilateral common carotid artery occlusion in mouse. Neuroscience.

[13] Akram A, Gibson CL, Grubb BD (2013). Neuroprotection mediated by the EP4 receptor avoids the detrimental side effects of COX2 inhibitors following ischaemic injury. Neuropharmacology.

[14] Nishimaki S, Seki K (1999). An imbalance between prostacyclin and thromboxane in relation to cerebral blood flow in neonates with maternal preeclampsia. Prostaglandins Other Lipid Mediat.

[15] Karasawa Y, Komiyama H, Yoshida S, Hino N, Katsuura Y, Nakaike S, Araki H (2003). Effect of TTC-909 on cerebral infarction following permanent occlusion of the middle cerebral artery in stroke prone spontaneously hypertensive rats. J Pharmacol Sci.

[16] Ohyama H, Hosomi N, Takahashi T, Mizushige K, Kohno M (2001). Thrombin inhibition attenuates neurodegeneration and cerebral edema formation following transient forebrain ischemia. Brain Res.

[17] de Leval X, Hanson J, David JL, Masereel B, Pirotte B, Dogné JM (2004). New developments on thromboxane and prostacyclin modulators part II: prostacyclin modulators. Curr Med Chem.

[18] Dogné JM, de Leval X, Hanson J, Frederich M, Lambermont B, Ghuysen A, Casini A, Masereel B, Ruan KH, Pirotte B, Kolh P (2004). New developments on thromboxane and prostacyclin modulators part I: thromboxane modulators. Curr Med Chem.

[19] Shohami E, Globus M, Weidenfeld J (1985). Regional distribution of prostanoids in rat brain: effect of insulin and 2-deoxyglucose. Exp Brain Res.

[20] Fang YC, Wu JS, Chen JJ, Cheung WM, Tseng PH, Tam KB, Shyue SK, Chen JJ, Lin TN (2006). Induction of prostacyclin/PGI2 synthase expression after cerebral ischemia-reperfusion. J Cereb Blood Flow Metab.

[21] Takechi H, Matsumura K, Watanabe Y, Kato K, Noyori R, Suzuki M, Watanabe Y (1996). A novel subtype of the prostacyclin receptor expressed in the central nervous system. J Biol Chem.

[22] Yagami T, Takahara Y, Ishibashi C, Sakaguchi G, Itoh N, Ueda K, Nakazato H, Okamura N, Hiramatsu Y, Honma T, Arimura A, Sakaeda T, Katsuura G (2004). Amyloid beta protein impairs motor function via thromboxane A2 in the rat striatum. Neurobiol Dis.

[23] Yokota C, Kaji T, Kuge Y, Inoue H, Tamaki N, Minematsu K (2004). Temporal and topographic profiles of cyclooxygenase-2 expression during 24 h of focal brain ishemia in rats. Neurosci Lett.

[24] Huttemeier PC, Kamiyama Y, Su M, Watkins WD, Benveniste H (1993). Microdialysis measurements of PGD2, TXB2 and 6-KETO-PGF1 alpha in rat CA1 hippocampus during transient cerebral ischemia. Prostaglandins.

[25] Cheng O, Li Z, Han Y, Jiang Q, Yan Y, Cheng K (2012). Baicalin improved the spatial learning ability of global ischemia/reperfusion rats by reducing hippocampal apoptosis. Brain Res.

[26] Kuang G, He Q, Zhang Y, Zhuang R, Xiang A, Jiang Q, Luo Y, Yang J (2012). Modulation of preactivation of PPAR-β on memory and learning dysfunction and inflammatory response in the hippocampus in rats exposed to global cerebral ischemia/reperfusion. PPAR Res.

[27] Wang H, Jiang R, He Q, Zhang Y, Zhang Y, Li Y, Zhuang R, Luo Y, Li Y, Wan J, Tang Y, Yu H, Jiang Q, Yang J (2012). Expression pattern of peroxisome proliferator-activated receptors in rat hippocampus following cerebral ischemia and reperfusion injury. PPAR Res.

[28] Song Y, Liu J, Zhang F, Zhang J, Shi T, Zeng Z (2013). Antioxidant effect of quercetin against acute spinal cord injury in rats and its correlation with the p38MAPK/iNOS signaling pathway. Life Sci.

[29] Whittaker N, Bunting S, Salmon J, Moncada S, Vane JR, Johnson RA, Morton DR, Kinner JH, Gorman RR, McGuire JC, Sun FF (1976). The chemical structure of prostaglandin X (prostacyclin). Prostaglandins.

[30] Brash AR, Jackson EK, Saggese CA, Lawson JA, Oates JA, FitzGerald GA (1983). Metabolic disposition of prostacyclin in humans. J Pharmacol Exp Ther.

[31] Patrono C, Ciabattoni G, Pugliese F, Pierucci A, Blair IA, FitzGerald GA (1986). Estimated rate of thromboxane secretion into the circulation of normal humans. J Clin Invest.

[32] Manzanero S, Santro T, Arumugam TV (2013). Neuronal oxidative stress in acute ischemic stroke: sources and contribution to cell injury. Neurochem Int.

[33] Ohsawa I, Ishikawa M, Takahashi K, Watanabe M, Nishimaki K, Yamagata K, Katsura K, Katayama Y, Asoh S, Ohta S (2007). Hydrogen acts as a therapeutic antioxidant by selectively reducing cytotoxic oxygen radicals. Nat Med.

[34] Kumar A, Mittal R, Khanna HD, Basu S (2008). Free radical injury and blood–brain barrier permeability in hypoxic-ischemic encephalopathy. Pediatrics.

[35] Wu KJ, Hsieh MT, Wu CR, Wood WG, Chen YF (2012). Green tea extract ameliorates learning and memory deficits in ischemic rats via its active component polyphenol epigallocatechin-3-gallate by modulation of oxidative stress and neuroinflammation. Evid Based Complement Alternat Med.

[36] Cherubini A, Ruggiero C, Polidori MC, Mecocci P (2005). Potential markers of oxidative stress in stroke. Free Radic Biol Med.

[37] Candelario-Jalil E, Mhadu NH, Al-Dalain SM, Martínez G, León OS (2001). Time course of oxidative damage in different brain regions following transient cerebral ischemia in gerbils. Neurosci Res.

[38] Yang J, Liu B, He B, Zhou Q (2006). Protective effects of meloxicam on aluminum overload-induced cerebral damage in mice. Eur J Pharmacol.

[39] Borre Y, Lemstra S, Westphal KG, Morgan ME, Olivier B, Oosting RS (2012). Celecoxib delays cognitive decline in an animal model of neurodegeneration. Behav Brain Res.

[40] Zhao Y, Patzer A, Herdegen T, Gohlke P, Culman J (2006). Activation of cerebral peroxisome proliferator-activated receptors gamma promotes neuroprotection by attenuation of neuronal cyclooxygenase-2 overexpression after focal cerebral ischemia in rats. FASEB J.

[41] Im JY, Kim D, Paik SG, Han PL (2006). Cyclooxygenase-2-dependent neuronal death proceeds via superoxide anion generation. Free Radic Biol Med.

[42] Lee J, Kosaras B, Aleyasin H, Han JA, Park DS, Ratan RR, Kowall NW, Ferrante RJ, Lee SW, Ryu H (2006). Role of cyclooxygenase-2 induction by transcription factor Sp1 and Sp3 in neuronal oxidative and DNA damage response. FASEB J.

[43] Cannon CP, Cannon PJ (2012). Physiology. COX2 inhibitors and cardiovascular risk. Science.

[44] Hao CM, Redha R, Morrow J, Breyer MD (2002). Peroxisome proliferator-activated receptor delta activation promotes cell survival following hypertonic stress. J Biol Chem.

[45] Saleem S, Shah ZA, Maruyama T, Narumiya S, Doré S (2010). Neuroprotective properties of prostaglandin I2 IP receptor in focal cerebral ischemia. Neuroscience.

[46] Matsuo Y, Izumiyama M, Onodera H, Kurosawa A, Kogure K (1993). Effect of a novel thromboxane A2 receptor antagonist, S-1452, on postischemic brain injury in rats. Stroke.

[47] Zhu HB, Zhang L, Wang ZH, Tian JW, Fu FH, Liu K, Li CL (2005). Therapeutic effects of hydroxysafflor yellow A on focal cerebral ischemic injury in rats and its primary mechanisms. J Asian Nat Prod Res.

[48] Marmol F, Puig-Parellada P, Sanchez J, Trullas R (1999). Influence of aging on thromboxane A2 and prostacyclin levels in rat hippocampal brain slices. Neurobiol Aging.

[49] Lundblad C, Grände PO, Bentzer P (2008). Increased cortical cell loss and prolonged hemodynamic depression after traumatic brain injury in mice lacking the IP receptor for prostacyclin. J Cereb Blood Flow Metab.

[50] Xiao CY, Hara A, Yuhki K, Fujino T, Ma H, Okada Y, Takahata O, Yamada T, Murata T, Narumiya S, Ushikubi F (2001). Roles of prostaglandin I(2) and thromboxane A(2) in cardiac ischemia-reperfusion injury: a study using mice lacking their respective receptors. Circulation.

[51] Cui Y, Kataoka Y, Satoh T, Yamagata A, Shirakawa N, Watanabe Y, Suzuki M, Yanase H, Kataoka K, Watanabe Y (1999). Protective effect of prostaglandin I(2) analogs on ischemic delayed neuronal damage in gerbils. Biochem Biophys Res Commun.

[52] Cui Y, Takamatsu H, Kakiuchi T, Ohba H, Kataoka Y, Yokoyama C, Onoe H, Watanabe Y, Hosoya T, Suzuki M, Noyori R, Tsukada H, Watanabe Y (2006). Neuroprotection by a central nervous system-type prostacyclin receptor ligand demonstrated in monkeys subjected to middle cerebral artery occlusion and reperfusion: a positron emission tomography study. Stroke.

[53] Hatae T, Wada M, Yokoyama C, Shimonishi M, Tanabe T (2001). Prostacyclin-dependent apoptosis mediated by PPAR delta. J Biol Chem.

[54] Fauti T, Müller-Brüsselbach S, Kreutzer M, Rieck M, Meissner W, Rapp U, Schweer H, Kömhoff M, Müller R (2006). Induction of PPARbeta and prostacyclin (PGI2) synthesis by Raf signaling: failure of PGI2 to activate PPAR beta. FEBS J.

[55] Gao H, Peng B, Welch WJ, Wilcox CS (1997). Central thromboxane receptors: mRNA expression and mediation of pressor responses. Am J Physiol.

[56] Honma S, Saika M, Ohkubo S, Kurose H, Nakahata N (2006). Thromboxane A2 receptor-mediated G12/13-dependent glial morphological change. Eur J Pharmacol.

[57] Wacker MJ, Tevis O, Hanke J, Howard T, Gilbert W, Orr JA (2012). Characterization of thromboxane A2 receptor and TRPV1 mRNA expression in cultured sensory neurons. Neurosci Lett.

[58] Wacker MJ, Tyburski JB, Ammar CP, Adams MC, Orr JA (2005). Detection of thromboxane A(2) receptor mRNA in rabbit nodose ganglion neurons. Neurosci Lett.

[59] Lin H, Li HF, Lian WS, Chen HH, Lan YF, Lai PF, Cheng CF (2013). Thromboxane A2 mediates iron-overload cardiomyopathy in mice through calcineurin-nuclear factor of activated T cells signaling pathway. Circ J.

